# IL-33 induces NF-κB activation in ILC2 that can be suppressed by *in vivo* and *ex vivo* 17β-estradiol

**DOI:** 10.3389/falgy.2022.1062412

**Published:** 2022-11-25

**Authors:** Shubhanshi Trivedi, Daniel Labuz, Cassandra E Deering-Rice, Chu Un Kim, Hayden Christensen, Sam Aamodt, Tom Huecksteadt, Karl Sanders, Kristi J. Warren

**Affiliations:** ^1^ Division of Infectious Disease, Department of Internal Medicine, University of Utah, Salt Lake City, UT, United States; ^2^ George E Wahlen Department of Veterans Affairs Medical Center, VA Salt Lake City Health Care System, Salt Lake City, UT, United States; ^3^ Department of Pharmacology and Toxicology, University of Utah College of Pharmacy, Salt Lake City, UT, United States; ^4^ Division of Pulmonary Medicine, Department of Internal Medicine, University of Utah Health, Salt Lake City, UT, United States

**Keywords:** asthma, allergy, ILC2 - group 2 innate lymphoid cell, estrogen, interleukin-5, interleukin-13, interleukin-33

## Abstract

Asthmatic women tend to develop severe airway disease in their reproductive years, and 30%–40% of asthmatic women have peri-menstrual worsening of asthma symptoms. This indicates that fluctuations in ovarian hormones are involved in advancement of asthmatic disease and exacerbation of symptoms. Group 2 innate lymphoid cells, or ILC2, are readily detected in allergic conditions, such as rhinosinusitis, in individuals that develop nasal polyps do to allergen exposures, and in allergic asthma. ILC2 are airway localized immune cells activated by IL-33, an innate cytokine that perpetuates allergic inflammation by driving the production of IL-5 and IL-13. We have previously shown that ILC2 are highly activated in naïve and ovalbumin (OVA) challenged, female BALB/c mice in comparison to male mice following stimulation with IL-33. Here, we investigated the effect of steady-state ovarian hormones on ILC2 and the NF-κB signaling pathway following OVA sensitization and challenge. We found that estrogen-treated ovariectomized mice (OVX-E2) that had been challenged with OVA had reduced IL-5 and IL-13 production by lung ILC2 as compared to lung ILC2 isolated from intact male and female sham-operated controls that had been treated with OVA. ILC2 were isolated from untreated animals and co-cultured *ex vivo* with and without estrogen plus IL-33. Those estrogen-treated ILC2 similarly produced less IL-5 and IL-13 in comparison to untreated, and had reduced NF-κB activation. Single-cell RNA sequencing showed that 120 genes were differentially expressed in male and female ILC2, and *Nfkb1* was found among top-ranked regulatory interactions. Together, these results provide new insight into the suppressive effect of estrogen on ILC2 which may be protective in female asthmatics. Understanding further how estrogen modulates ILC2 may provide therapeutic targets for the treatment of allergic diseases.

## Introduction

Asthma is a chronic respiratory disease associated with high levels of type 2 inflammation. Notably, asthma prevalence, hospitalizations, and mortality are higher among women than men of reproductive age (1, 2). However, the opposite is true for children as more boys than girls have asthma and are hospitalized due to asthma-related complications (3–5). This change that occurs around the time of puberty, implicates sex hormones as a contributor to this sex-bias in asthma. Indeed, about 30–40% of asthmatic women experience peri-menstrual worsening of asthma (6), and 50% of those women are hospitalized because of a peri-menstrual related exacerbations (7). The role of estrogen in this phenomenon remains a subject of debate with most suggesting pro-inflammatory activity to estrogen (8–11) and others showing a protective role (12, 13). Additional studies need to determine mechanisms by which sex hormones regulate asthma symptoms.

Lung resident eosinophils, macrophages, type 2 helper T (Th2) cells and group 2 innate lymphoid cells (ILC2) are activated directly by IL-33. IL-33 is a constitutively expressed innate cytokine that is released from EpCAM+ airway epithelial cells to promote type 2 inflammatory reactions. We have shown an increased activation in female lung ILC2 treated with IL-33 compared to male BALB/c mice following *ex vivo* stimulation with IL-33 (14). IL-33 activates NF-κB in a wide variety of immune cells (15). NF-κB is composed of a family of transcription factors that control various aspects of the immune system and inflammatory responses. In most immune cells, NF-κB is present as a latent, inactive dimer bound to the inhibitory IκB proteins in the cytoplasm. Activation of the NF-κB dimers occurs as a result of IκB kinase (IKK)-mediated, phosphorylation-induced degradation of the IκB inhibitor. This degradation enables the NF-κB dimers to enter the nucleus and activate specific target genes such as IL-5 and IL-13 leading to their expression following IL-33 stimulation (16, 17). NF-κB signals through distinct canonical and non-canonical pathways. In the canonical pathway, IκB is phosphorylated by IKKβ and NF-κB essential modulator (NEMO), which results in the nuclear translocation of RelA, c-Rel and p50 homo- and heterodimers. In contrast, the non-canonical pathway involves IKKα-mediated phosphorylation of p100 and association with RelB, which leads to partial processing of p100 and translocation of transcriptionally active p52-RelB complexes to the nucleus (18).

Estrogen can directly and indirectly effect the NF-κB signaling pathway through mutually antagonistic cross-talk between NF-κB and the estrogen receptor (ER) signaling pathways. ERα has been shown to inhibit NF-κB activity in an estrogen dependent manner in various cell lines, ERβ also has been shown to have an inhibitory effect on NF-κB activity (19–21). ERs can inhibit NF-κB activity by various mechanisms, either by acting in the cytoplasm to modify upstream NF-κB signaling, or they can act in the nucleus through DNA binding or transcriptional activation (19, 22). As an example, studies identified ER as one protein capable of binding to the p65 subunit of NF-κB, and when bound to DNA this interaction was inhibitory for ER transcriptional activity (23). Inhibition of NF-κB activity by estrogen is attributed to effects of ER on IkB processing which itself inhibits DNA binding and nuclear translocation of NF-κB (24, 25). Although the cross-talk between NF-κB and ER transcription factors has been shown in various studies the molecular mechanisms seem to be complex and specific to cell type and context. At present, there is a paucity of data to clarify the role of 17β-estradiol (E2) and/or ERs in regulating the activity of NF-κB in ILC2.

E2 treatment reduces ILC2 accumulation, and IL-5 and IL-13 cytokine responses, in the lung and airways of ovalbumin (OVA)-challenged ovariectomized (OVX) mice. In this study, OVX animals were similarly given steady-state E2 then challenged with OVA to more intricately investigate the interaction between estrogen and NF-κB activation in lung-localized ILC2. We show that estrogen reduces NF-κB activity in males and in females. Expanding our strategies for reducing IL-5 and IL-13 cytokine production by ILC2 can improve the allergy and asthma phenotype in men and women.

## Materials and methods

### Animals, *in vivo* hormones and allergen challenges

Eight-week old, male and female, BALB/c mice (Charles River, O’Fallon, MO) were acclimated at our facility for at least 1 week prior to experimental procedures. All mice had *ad libitum* access to rodent chow and filtered water throughout the course of the experiment. Female BALB/c mice were ovariectomized at 3 weeks of age. Three weeks old male and female animals underwent sham surgery and were included as controls. Mice were transported to the Salt Lake City VA Medical Center (Salt Lake City, UT) after 2 weeks of recovery from surgery. Animals were acclimated for 1 week, before hormone placement. Ovariectomized mice were deeply anesthetized with ketamine (80 mg/kg) and xylazine (8 mg/kg) then implanted with a subcutaneous pellet containing estrogen (E2; 0.1 mg) (OVX-E2) (Innovative Research of America, Sarasota, FL, USA) between the scapula delivering 60-day steady release of E2. We evaluated ILC2 responses in male and female sham operated and OVX-E2 animals comparatively. OVX mice which received placebo pellet were excluded from the study because we found no statistical difference between OVX-placebo OVA mice and female sham OVA mice in our previous studies evaluating allergic outcomes (26). Post-surgery animals were given two doses of buprenorphine (0.05 mg/kg) 12 h apart for pain management. Two weeks later animals were sensitized with ovalbumin (500 µg/ml) (Grade V; Sigma, St. Lious, MO) and aluminum hydroxide (20 mg/ml) (Sigma) once per week for 3 weeks. Intranasal OVA challenges (1.5% in sterile saline) were initiated 1 week later and given daily for 5 consecutive days (14). For single-cell sequencing experiments male and female BALB/c were challenged for 4 consecutive days with intranasal *Alternaria alternata* extract (5 μg/mouse in 50 μl) (Greer, Lenoir, NC). All animals were euthanized using ketamine overdose (200 mg/kg). All protocols were approved by the IACUC and Research Advisory Committees at the Salt Lake City VA Medical Center.

### Preparation of single cell suspension and flow cytometry

Lung tissues were harvested and subjected to an automated dissociation procedure using a gentleMACS Dissociator according to the manufacturer's instructions (Miltenyi Biotech, Auburn, California) in a solution containing collagenase type I (324 U/ml; Fisher, Pittsburgh, PA) and DNAse (25 U/ml, Fisher). The suspension was incubated for an additional 15–30 min at 37°C to facilitate further breakdown of collagen in the lung. To remove any large fragments, the cell solution was passed through nylon mesh (40 μM; Fisher), the strainer was rinsed with 5–10 ml of PBS and cells were centrifuged at 350 × *g* for 5 min. The resulting cell pellet was resuspended in 2 ml of ACK Lysing Buffer (Thermo Fisher Scientific, USA), centrifuged and resuspended with EasySep Buffer (STEMCELL Technologies Vancouver, Canada). Viability was assessed by trypan blue exclusion on the TC-20 cell counter (Biorad, Hercules, CA).

**Figure 1:**
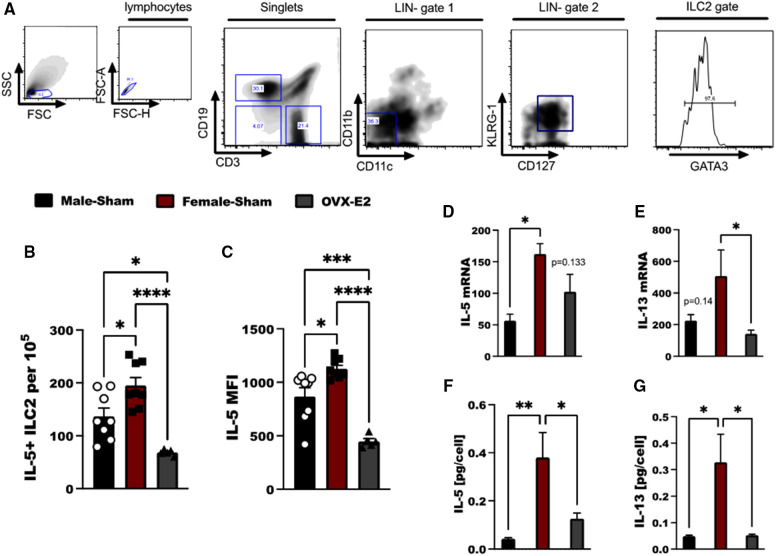
E2 treatment reduces IL-33 mediated IL-5 and IL-13 expression in ILC2. (**A**) Flow cytometry gating strategy of ILC2 defined as live, singlet cells lacking lineage markers (CD11b, CD11c) and expressing KLRG-1, CD127 and GATA3. Cytokine expression by ILC2 in Male-sham, female-sham, and estrogen-treated OVX mice is shown. (**B,C**) Concentration of IL-5-producing ILC2 and the mean fluorescence intensity of IL-5 measured by flow cytometry. (**D,E**) *il5* and *il13* mRNA expression by ILC2 measured by qRT-PCR. (**F,G**) IL-5 and IL-13 protein expression measured by ELISA. Error bars denote means ± SEM (*n* = 6–8 mice/group). Data from two experiments are shown. Groups were compared using one-way ANOVA followed by Tukey's multiple comparisons test. *p < 0.05; **p < 0.01; ***p < 0.001; ****p < 0.0001.

Cell suspensions from each animal were incubated with anti-CD16/32 (Fc Block, BD Biosciences, San Jose, CA) to minimize non-specific antibody staining and surface stained with anti-mouse antibodies against mouse lineage antibody cocktail (clone 145–2C11, which recognizes Mouse CD3e; M1/70, which recognizes CD11b; RA3–6B2, which recognizes CD45R/B220; TER-119, which recognizes Ly-76, mouse erythroid cells; and RB6–8C5, which recognizes Ly-6G and Ly-6C.), and CD3, CD19, CD11c, CD11b, CD45, CD127 and KLRG-1. This was followed by intracellular cell staining with anti-mouse antibodies against GATA-3, IL-5, IL-13 and phospho-p65 (BD Biosciences, San Jose, CA) using a FOXP3 kit (BD Bioscience). The staining was performed according to the manufacturer's instructions. Parallel cell preparations were treated with appropriate isotype controls. Cytometer compensation was performed with antibody capture beads (eBioscience, Grand Island, NY) stained separately with individual antibodies used in test samples. Lymphocyte populations were gated by characteristic forward and side scatter properties and antibody-specific staining fluorescence intensity using a FACSAria (BD Biosciences) with gating strategy depicted in Figure 1A.

### Lung ILC2 cell isolation and activation

Lung tissue was dissociated and ILC2 enrichment was performed with the EasySep Mouse ILC2 Enrichment Kit (STEMCELL Technologies) according to manufacturer instructions. Cells were labeled with antibodies to common immune lineage markers, as described in the previous section. Gating was set on a FACS Aria (BD Biosciences) to acquire ILC2 cells defined as CD45+ LIN− cells that co-express CD127 and KLRG-1. ILC2 cells were sorted by flow cytometry using FACS Aria II system from BD Biosciences (San Jose, California). 2–5 × 10^3^ sorted ILC2 were added to each well of a 96 well U bottom plate followed by pretreatment with E2 (25 or 2.5 pg/ml) for 1 h in 37°C incubator. Finally, ILC2 were cultured with IL-2 (10 ng/ml); IL-7 (10 ng/ml); and IL-33 (10 ng/ml). Culture conditions were maintained for 4–5 days. After culture, cells were separated from supernatants by centrifugation at 400 × *g* for 10 min. Supernatants were stored at −80°C until further analysis was completed.

### Enzyme-linked immunosorbent assay (ELISA)

Cell culture supernatants were centrifuged at 400 × *g* at room temperature for 10 min to clear cellular debris prior to ELISA testing. IL-5 (assay range: 31.2–2,000 pg/ml) and IL-13 (assay range: 62.5–4,000 pg/ml) Duo-set ELISA (R&D Systems, Minneapolis, MN) were performed according to the manufacturer's instructions. ILC2 culture supernatants were diluted at least 1:2–1:10 with reagent diluent provided with the Ancillary Reagents Kit II (R&D Systems) that accompanies the ELISA Duo-set kits. Absorbence was measured on the SpectraMax M5e (VWR, Radnor, PA) or the Clariostar (BMG LABTECH, Ortenberg, Germany) at 450 nm with 570 nm wavelength correction.

### RNA isolation and polymerase chain reaction

RNA was collected from cell lysates using the ZymoResearch MicroPrep kit (Genesee Scientific, San Diego, California). 10–100 ng of RNA was converted to cDNA using the qPCRBIO cDNA Synthesis kit from Genesee according to the manufacturer's guidelines for small amounts of RNA. The product of the cDNA reaction was diluted 1:3 with DNase/RNase-free water (Sigma Aldrich, St. Louis, Missouri, US) and applied to a 20 μl PCR reactions using the pre-designed primers from Sigma Aldrich (St. Louis, Missouri, US) that accompany the KicQ Start SYBR Green Master Mix (Sigma), using pre-determined annealing and amplification cycling, up to 40 cycles. Given the small quantity of RNA available applied to cDNA reactions each cDNA product was sufficient to run 5–6 gene targets in duplicate, in addition to the HK gene (GAPDH). PCR was performed on the ABI 7500 under fast cycling conditions, Ct values were determined and subsequent analysis was performed using the software StepOne Real-Time PCR System (ABI 7500). The delta delta Ct method was used to calculate fold-change of the various treatment condition to IL-2/IL-7 stimulated cells, or baseline.

### Single-cell RNA-seq analysis

Lung ILC2 (CD45+ LIN− CD127+ KLRG-1+) were sorted from male and female *Alternaria alternata* treated BALB/c mice using BD FACS Aria III Cell sorter. Cells were incubated with BD AbSeq Ab-oligos CD19, CD11c, CD25, CD117, IL-33R, CD278 and CD25 to further differentiate ILC2 for targeted single-cell RNA-seq analysis which was completed on the BD Rhapsody Single-Cell Analysis System according to the manufacturer's instructions. mRNA targeted, sample Tag, and BD™ AbSeq library preparation was completed using the BD Rhapsody™ targeted mRNA and AbSeq amplification kits and protocol. The final libraries of the AbSeq and mRNA were analyzed using Agilent 2200 TapeStation. The AbSeq-oligos, Sample Multiplex Tags, and ILC2 mRNA targeted libraries were pooled together before sequencing on a NovaSeq6000 instrument (Illumina). The FASTQ files were uploaded to Seven Bridges Genomics (NCBI BioSample accessions: SAMN31323861, SAMN31323862), and a workflow designed by BD Biosciences was used to analyze the data to demultiplex, identify cells based on AbSeq-oligo, and analyze the single-cell mRNA data. Analysis of the final count matrix was done through SeqGeq v1.70 (Becton Dickinson & Company) and Seurat v4.0.3 in R v4.1.2. Analysis of gene ontology biological process of top 20 differentially expressed genes (DEG), and pathway analysis of top 100 DEG between male and female ILC2 was done using Bioplanet tool in Enrichr. We used TRRUST (transcriptional regulatory relationships unraveled by sentence-based text-mining) analysis on top 100 DEG in metascape tool as in previous publications (27–30).

### Statistical analysis

Data are presented as the mean ± standard error of mean (SEM). Statistics were performed using one-way analysis of variance (ANOVA) with Tukey and Dunnett's multi-comparison post-test was employed to compare differences among 3 or more treatment groups, two-way ANOVA followed by Tukey's multiple comparison test was used to compare male vs. female mice and different treatment groups using GraphPad (version 5.01) software. In all analyses, *p* values less than 0.05 were considered statistically significant.

## Results

### E2 treatment reduces Il-5 production by lung ILC2 in OVX mice sensitized and challenged with OVA

Lung ILC2 were isolated from OVX animals that had been implanted with an E2 pellet (OVX-E2), intact male-sham operated animals (M-Sham) and intact female sham-operated animals (F-Sham); all animals were sensitized and challenged with 5 days of intranasal OVA to generate substantial IL-33-driven allergic inflammation in the lungs (31). ILC2 were identified as live lymphocytes that are lineage negative (LIN-), but express KLRG-1, CD127 and GATA3 (Figure 1A). Following *ex vivo* stimulation with IL-33, we found increased numbers of IL-5+ ILC2 in female, sham-operated animals as compared to male, sham-operated animals following OVA stimulation (*p* < 0.05). OVX-E2 mice treated with OVA, however, had reduced IL-5 production in ILC2 compared to both intact male and female mice (*p* < 0.001 for comparison to females, and *p* < 0.05 as compared to males (Figures 1B,C). Furthermore, *il5* and *il13* gene expression were measured in lung ILC2 after *ex vivo* stimulation with IL-2, IL-7, and IL-33. When comparing lung ILC2 isolated from F-sham and OVX-E2 a significant reduction in *il13* transcripts were detected by qRT-PCR (*p* < 0.05); the reduction in *il5* transcripts did not reach statistical significance (*p* = 0.133) (Figures 1D,E). IL-5 and IL-13 protein secretion by lung ILC2 cells was also reduced in OVX-E2 mice compared to female control mice (*p* < 0.05) (Figures 1F,G).

### E2 treatment reduces NF-κB pathway components in ILC2

**Figure 2:**
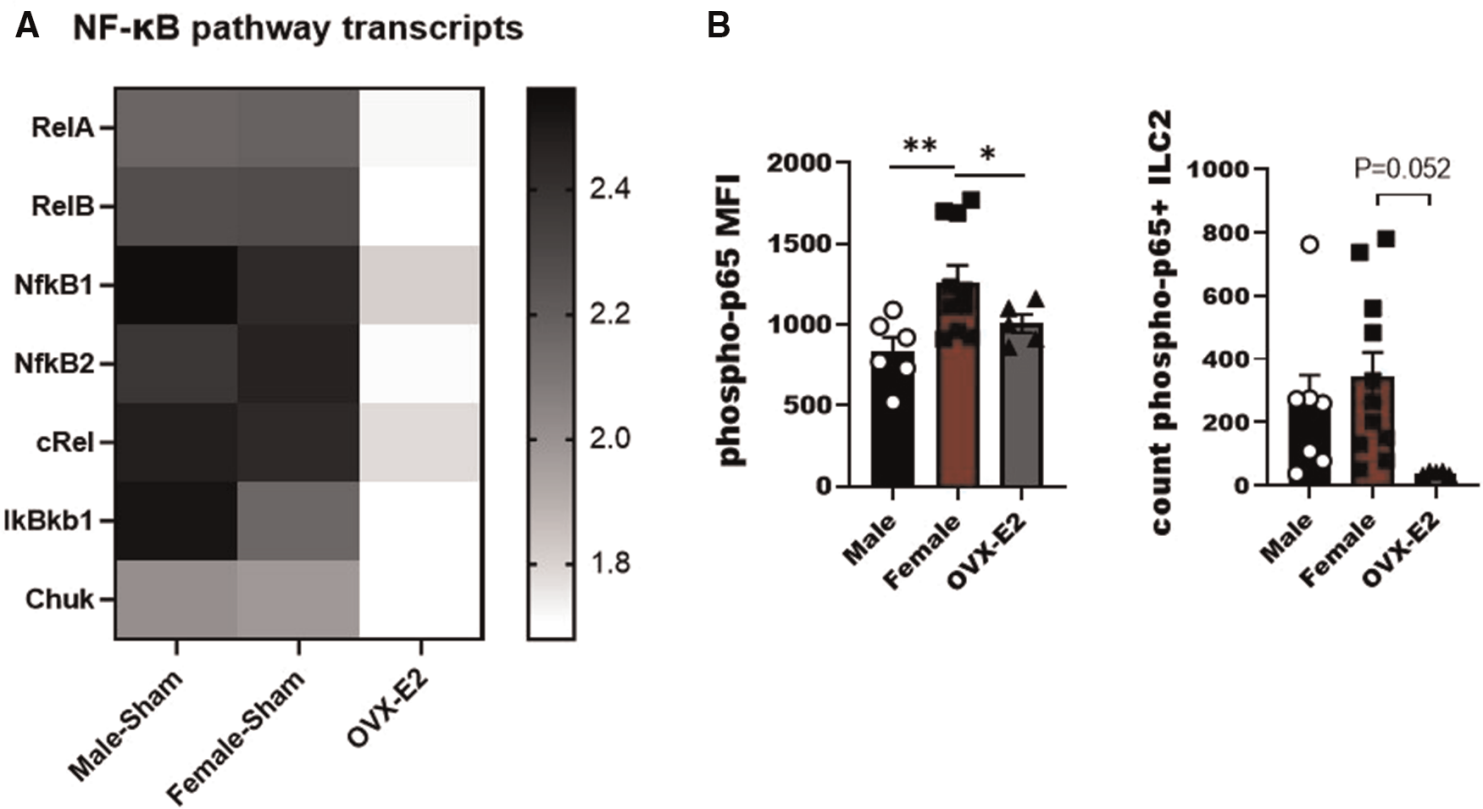
Broad screening of mRNA levels of mediators of the NF-κB pathway in E2-treated ILC2. ILC2 were sorted from the lungs of Male, female, and estrogen-treated OVX mice that had been sensitized and challenged with OVA (OVX-E2). Purified ILC2 were cultured with IL-2, IL-7 and IL-33 for 5 days and NF-κB pathway components gene expression was measured using qRT-PCR. (**A**) Heatmap shows fold-change gene expression of *relA*, *relB*, *NFκB1*, *NFκB2*, *cRel*, *IkBkb1* and *chuk*. (**B**) Counts of ILC2 with phosphorylated p65 and the mean fluorescence intensity of phosphorylated p65 measured by flow cytometry. Groups were compared using one-way ANOVA followed by Tukey's multiple comparisons test. *p < 0.05; **p < 0.01; ***p < 0.001; ****p < 0.0001.

IL-33 activates the NF-κB pathway in a MyD88-dependent manner to produce IL-5 and IL-13 by lymphocytes (32, 33). As such we developed a small panel of NF-κB associated genes to measure in ILC2 following stimulation with IL-33. We observed differences between tested groups in the members of canonical and non-canonical NF-κβ activation pathway, namely *relA*, *relB*, *Nfκβ1*, *Nfκβ2*, *cRel*, *IκBκβ1I* and *Chuk* genes. Most importantly, most of the canonical and non-canonical NF-κB genes were decreased in ILC2 isolated from OVX-E2 treated animals (Figure 2A). We also evaluated phosphorylation of the p65 subunit in ILC2 and definitively show that E2 suppresses the phosphorylation of p65 In OVX-E2 treated animals compared to F-Sham operated animals (*p* < 0.052) (Figure 2B).

**Figure 3:**
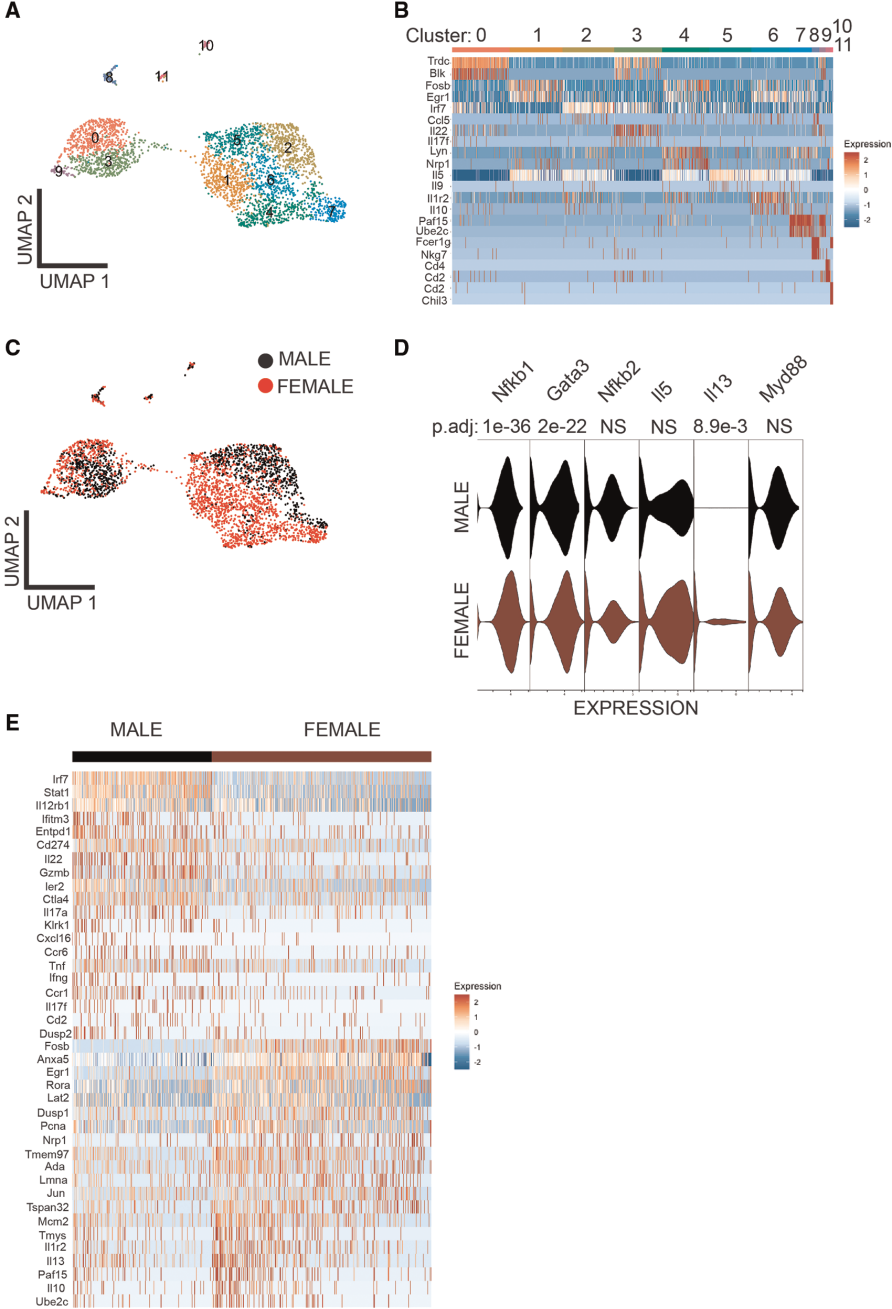
Single cell-RNA seq reveals differentially expressed genes in Male and female lung ILC2. (**A**) UMAP visualization shows 11 clusters of ILC2, (**B**) heat map shows top two DEG in each cluster (**C**) UMAP visualization shows sex differences in lung ILC2, (**D**) Violin plot of *myd88*, *il13*, *il5*, *nfkb2*, *gata3* and *nfkb1 I* genes, and (**E**) heat map of top 20 highly significantly differentially expressed genes in Male and female lung ILC2.

**Figure 4:**
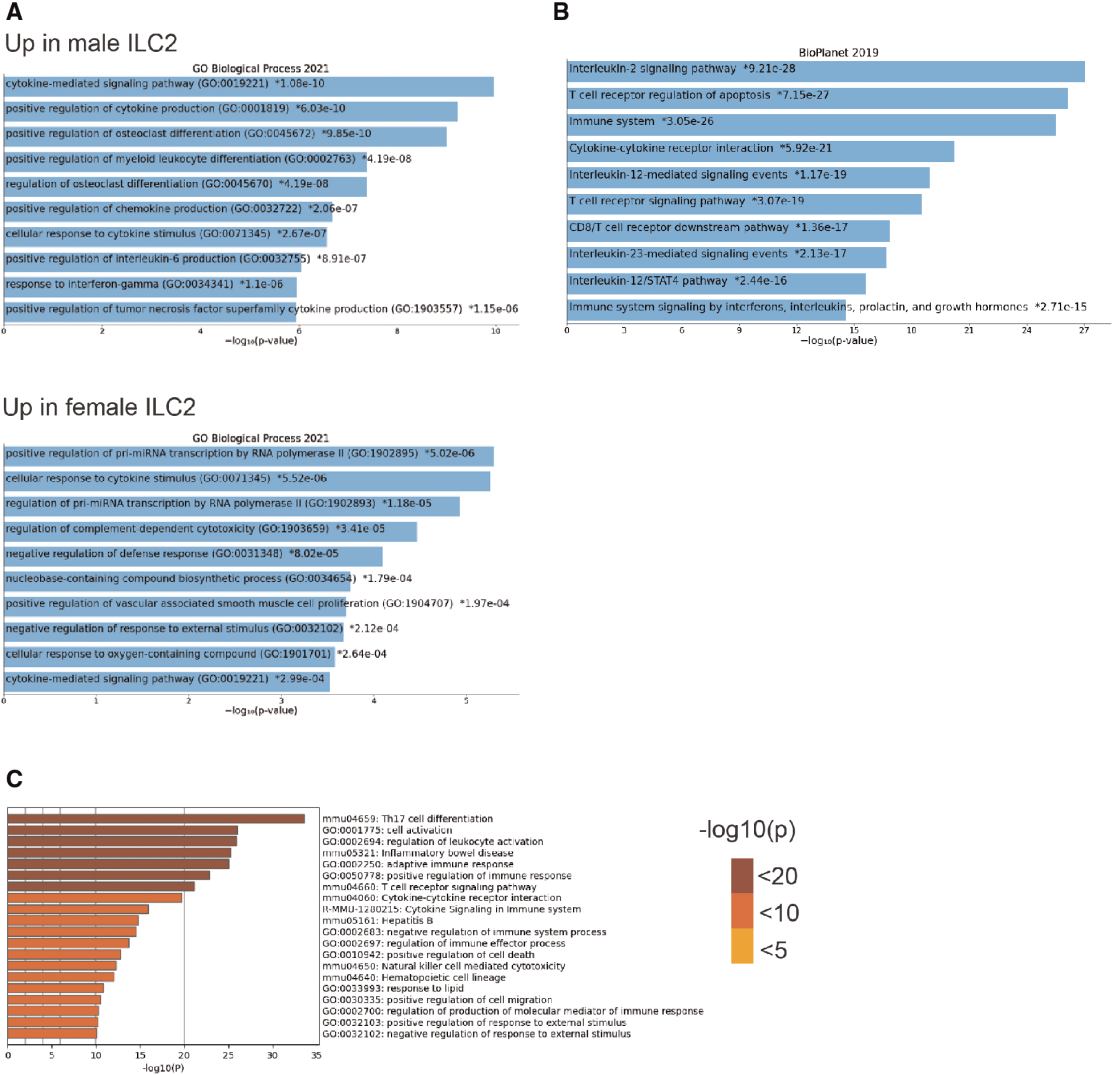
Gene ontology, pathway and TRRUST analysis reveals differences in male and female ILC2 (**A**) gene ontology analysis of top 20 DEG in male and female ILC2 using enrichr GO biological process 2021, (**B**) bioplanet pathway analysis of top 100 DEG using enrichr (**C**) TRRUST analysis of top 100 DEG in male and female ILC2 isolated following challenge with *alternaria* using metascape, bar graph are sorted by *p*-value ranking.

To understand the sex-bias in asthma we performed targeted single-cell RNA sequencing (scRNA-seq) on male and female ILC2 sorted from the lungs of *Alternaria alternata* treated animals. Although OVA has been used in experimental models of asthma and induces intense allergic pulmonary inflammation, for single-cell sequencing experiment we decided to use *Alternaria alternate* mouse model because of several limitations of OVA model such as OVA is not a natural allergen for human asthma and its long term challenge might lead to the development of tolerance (34–36). Dimensional reduction and visualization using Uniform Manifold Approximation and Projection (UMAP) demonstrated that there are eleven different populations of ILC2 (Figure 3A). The top two DEG in cluster 1, 4 and 6, which largely consisted of female ILC2, include *fosb* and *egr1* (cluster 1), *lyn* and *nrp1* (cluster 4) and *il1r2* and *il10* (cluster 6) genes (Figure 3B). We found that male and female ILC2 cluster separately based on gene expression profiles (Figure 3C), out of 357 genes analyzed 120 genes were differentially expressed in male and female ILC2 (Table 1). Violin plots showed differential expression of *nfkb1*, *gata3*, and *il13* genes in male and female ILC2 (Figure 3D). When we look at top 20 DEG (Figure 3E), we found lung ILC2 from male mice have higher expression of *Irf7*, *Stat1*, *il12rb1*, *Ifitm3*, *Entpd1*, *Cd274*, *il22*, *gzmB*, *ler3*, *ctla4*, *il17a*, *klrk1*, *cxcl16*, *ccr6*, *tnf*, *ifng*, *ccr1*, *il17f*, *cd2* and *dusp2* genes and lower expression of *fosb*, *anxa5*, *egr1*, *rora*, *lat2*, *dusp1*, *pcna*, *nrp1*, *tmem97*, *ada*, *lmna*, *jun*, *tspan32*, *mcm2*, *tyms*, *il1r2*, *il13*, *2810417h13r1k*, *il10* and *ubec2c* genes compared to female mice. Consistent with our previous findings we found that female ILC2 show significant upregulation of *il13* [log2 fold change (L_2_FC) = −0.856, *p*.adj < 0.0088], *Nfkb1* (L_2_FC = −0.5268, *p*.adj < 1.36 × 10^−36^) and *Gata3* (L_2_FC = −0.5885, *p*.adj < 2.54 × 10^−22^) compared to male ILC2 (Table 1 and Figures 3D,E). Next, to identify the functionality of top 20 DEG in male and female ILC2 we performed gene ontology analysis using gene ontology biological process tool in Enrichr (29). We found positive regulation of cytokine and chemokine production in males and positive regulation of vascular associated smooth muscle cell proliferation in females (Figure 4A). When pathway analysis was performed in top 100 DEG (Table 1) between male and female ILC2 using BioPlanet tool in Enrichr, we found that IL-2 signaling, cytokine-cytokine receptor interactions and signaling by interleukins, prolactin and growth hormones significantly differ between male and female ILC2 (Figure 4B). TRRUST (transcriptional regulatory relationships unraveled by sentence-based text-mining) analysis in metascape tool (37, 38), found *Nfkb1* among top-ranked regulatory interactions (Figure 4C). These results confirm the sex differences for ILC2 and involvement of NF-κB pathway components in intranasal *Alternaria* challenged male and female mice.

**Table 1:** Differentially expressed genes in male and female ILC2.

Genes	*p*_val	avg_log2FC (male vs. female)	pct.1 (% male ILC2 expressing gene)	pct.2 (% female expressing gene)	*p*_val_adj
Fosb	2.11 × 10^−143^	−3.515030041	0.123	0.505	7.52 × 10^−141^
Anxa5	1.61 × 10^−136^	−1.23737979	0.928	0.949	5.75 × 10^−134^
Irf7	4.17 × 10^−126^	1.459953569	0.748	0.342	1.49 × 10^−123^
Stat1	7.42 × 10^−86^	1.061494968	0.776	0.46	2.65 × 10^−83^
Gapdh	7.10 × 10^−65^	−0.445734056	0.999	1	2.53 × 10^−62^
Egr1	8.54 × 10^−65^	−1.536078568	0.284	0.519	3.05 × 10^−62^
S100a10	4.35 × 10^−56^	−0.637064402	0.962	0.966	1.55 × 10^−53^
Lgals3	6.43 × 10^−54^	−0.565533184	0.941	0.954	2.29 × 10^−51^
Itgb2	3.89 × 10^−51^	−0.628946638	0.864	0.873	1.39 × 10^−48^
Rora	7.98 × 10^−48^	−0.929468928	0.562	0.641	2.85 × 10^−45^
Hprt	1.47 × 10^−43^	−0.612686879	0.703	0.713	5.24 × 10^−41^
Zap70	1.99 × 10^−43^	−0.589689998	0.849	0.851	7.09 × 10^−41^
Il12rb1	7.07 × 10^−43^	0.639472814	0.796	0.578	2.52 × 10^−40^
Lat2	7.53 × 10^−43^	−0.811020397	0.577	0.68	2.69 × 10^−40^
H2-K1	7.89 × 10^−40^	0.303138833	1	1	2.82 × 10^−37^
Nfkb1	3.82 × 10^−39^	−0.526842463	0.857	0.844	1.36 × 10^−36^
Ifngr1	5.11 × 10^−39^	0.447694232	0.906	0.742	1.82 × 10^−36^
Icos	3.90 × 10^−38^	0.456779327	0.909	0.763	1.39 × 10^−35^
Ifitm3	2.41 × 10^−36^	1.40127314	0.221	0.066	8.61 × 10^−34^
Tnfrsf9	2.96 × 10^−36^	0.467910645	0.926	0.792	1.06 × 10^−33^
Entpd1	3.93 × 10^−33^	0.911021956	0.261	0.1	1.40 × 10^−30^
Cd274	2.60 × 10^−29^	0.781511169	0.488	0.29	9.26 × 10^−27^
Lamp1	3.38 × 10^−29^	−0.451801204	0.755	0.756	1.21 × 10^−26^
Ptprc	1.33 × 10^−28^	−0.340070653	0.952	0.921	4.75 × 10^−26^
Cd3e	2.25 × 10^−28^	0.366084583	0.371	0.219	8.02 × 10^−26^
Xbp1	7.66 × 10^−28^	−0.371763368	0.927	0.898	2.74 × 10^−25^
Dusp1	2.24 × 10^−27^	−1.255120697	0.234	0.344	8.01 × 10^−25^
Il22	1.31 × 10^−26^	2.193050105	0.231	0.109	4.68 × 10^−24^
Mapk1	1.73 × 10^−26^	−0.405573855	0.493	0.469	6.17 × 10^−24^
Pcna	2.77 × 10^−26^	−0.897958758	0.529	0.613	9.89 × 10^−24^
Il7r	6.08 × 10^−26^	0.50213201	0.532	0.342	2.17 × 10^−23^
Gzmb	6.20 × 10^−26^	1.56897527	0.369	0.222	2.21 × 10^−23^
Nrp1	1.17 × 10^−25^	−1.455967456	0.07	0.185	4.17 × 10^−23^
Tmem97	3.65 × 10^−25^	−0.991004103	0.244	0.341	1.30 × 10^−22^
Ier3	5.34 × 10^−25^	0.589208989	0.645	0.456	1.91 × 10^−22^
Selplg	6.02 × 10^−25^	−0.34536528	0.976	0.959	2.15 × 10^−22^
Tgfb3	6.23 × 10^−25^	0.521269095	0.605	0.416	2.22 × 10^−22^
Gata3	7.11 × 10^−25^	−0.58849849	0.69	0.774	2.54 × 10^−22^
Il18rap	2.86 × 10^−24^	−0.79707987	0.455	0.538	1.02 × 10^−21^
Ada	2.20 × 10^−23^	−1.005766403	0.209	0.309	7.84 × 10^−21^
Arg1	6.89 × 10^−23^	−0.542232712	0.626	0.77	2.46 × 10^−20^
Lmna	4.59 × 10^−21^	−1.198602364	0.131	0.22	1.64 × 10^−18^
Il3ra	3.30 × 10^−20^	0.583470777	0.278	0.144	1.18 × 10^−17^
Ikbkb	4.50 × 10^−20^	−0.533951329	0.413	0.458	1.61 × 10^−17^
Mbp	4.94 × 10^−20^	−0.692299497	0.391	0.464	1.76 × 10^−17^
Jun	1.41 × 10^−19^	−1.001443573	0.318	0.369	5.02 × 10^−17^
Vps28	2.01 × 10^−19^	−0.334299995	0.789	0.772	7.18 × 10^−17^
Bin2	5.66 × 10^−19^	−0.317812685	0.689	0.663	2.02 × 10^−16^
Ctla4	9.93 × 10^−19^	0.882109522	0.463	0.309	3.54 × 10^−16^
Ctsd	1.75 × 10^−18^	−0.275066941	0.918	0.918	6.26 × 10^−16^
Cd3d	3.08 × 10^−18^	0.520890066	0.324	0.191	1.10 × 10^−15^
Tspan32	1.01 × 10^−17^	−0.955968713	0.169	0.259	3.59 × 10^−15^
Atf6b	3.39 × 10^−17^	−0.363986475	0.396	0.359	1.21 × 10^−14^
Il2ra	3.50 × 10^−17^	−0.385504376	0.942	0.921	1.25 × 10^−14^
Il17a	5.24 × 10^−17^	1.585639642	0.205	0.102	1.87 × 10^−14^
Tnfsf14	3.20 × 10^−16^	−0.723452	0.23	0.3	1.14 × 10^−13^
Itk	4.49 × 10^−16^	−0.282261507	0.792	0.735	1.60 × 10^−13^
Igbp1	7.39 × 10^−16^	−0.387135735	0.425	0.423	2.64 × 10^−13^
Ccr5	1.10 × 10^−15^	0.537725966	0.493	0.347	3.94 × 10^−13^
Cd3g	1.16 × 10^−15^	0.35213825	0.382	0.252	4.13 × 10^−13^
Fyn	1.33 × 10^−15^	−0.273889281	0.53	0.508	4.75 × 10^−13^
Cd52	2.11 × 10^−15^	−0.35636077	0.755	0.744	7.52 × 10^−13^
Fas	2.97 × 10^−15^	0.486676129	0.278	0.16	1.06 × 10^−12^
Cd44	6.11 × 10^−15^	−0.376822285	0.641	0.653	2.18 × 10^−12^
Trbc1	1.06 × 10^−14^	−0.399150476	0.508	0.618	3.78 × 10^−12^
Klrk1	1.99 × 10^−14^	0.65915746	0.1	0.033	7.09 × 10^−12^
Bcl2	2.99 × 10^−14^	−0.476918324	0.442	0.494	1.07 × 10^−11^
Mcm2	9.98 × 10^−14^	−0.827703513	0.266	0.324	3.56 × 10^−11^
Vegfa	1.23 × 10^−13^	−0.560056391	0.386	0.43	4.40 × 10^−11^
Mcm4	1.33 × 10^−13^	−0.717022051	0.348	0.382	4.76 × 10^−11^
Socs1	2.19 × 10^−13^	0.389607561	0.502	0.366	7.84 × 10^−11^
Il23r	6.73 × 10^−13^	0.538497442	0.109	0.043	2.40 × 10^−10^
Lap3	1.80 × 10^−12^	−0.654405555	0.13	0.178	6.43 × 10^−10^
Lyn	2.74 × 10^−12^	−0.744898703	0.193	0.237	9.80 × 10^−10^
Stat6	5.57 × 10^−12^	−0.504070219	0.273	0.295	1.99 × 10^−9^
Tyk2	1.17 × 10^−11^	−0.340714932	0.414	0.422	4.18 × 10^−9^
Lat	1.38 × 10^−11^	−0.476166178	0.645	0.699	4.93 × 10^−9^
Cxcl16	1.76 × 10^−11^	0.600325933	0.058	0.014	6.28 × 10^−9^
Rorc	3.42 × 10^−11^	0.43342693	0.194	0.111	1.22 × 10^−8^
Ccr6	4.52 × 10^−11^	1.263050871	0.108	0.045	1.61 × 10^−8^
Tyms	4.63 × 10^−11^	−0.879531084	0.118	0.167	1.65 × 10^−8^
Itga4	6.17 × 10^−11^	−0.644568884	0.201	0.261	2.20 × 10^−8^
Cst7	2.06 × 10^−10^	−0.320964212	0.384	0.361	7.34 × 10^−8^
Mapk8	8.55 × 10^−10^	−0.58105315	0.141	0.185	3.05 × 10^−7^
Ybx3	1.01 × 10^−9^	−0.59735864	0.191	0.24	3.59 × 10^−7^
Bach2	1.63 × 10^−9^	−0.77265348	0.039	0.074	5.81 × 10^−7^
Tnfrsf8	1.63 × 10^−9^	0.258089865	0.538	0.426	5.82 × 10^−7^
Glg1	1.73 × 10^−9^	−0.250369105	0.297	0.288	6.18 × 10^−7^
Tnf	1.81 × 10^−9^	0.602835996	0.336	0.24	6.45 × 10^−7^
Ifng	5.83 × 10^−9^	1.328213682	0.08	0.031	2.08 × 10^−6^
Ccr1	6.00 × 10^−9^	0.709083965	0.175	0.1	2.14 × 10^−6^
Il1r2	1.08 × 10^−8^	−0.969717618	0.183	0.257	3.85 × 10^−6^
Cd37	1.96 × 10^−8^	−0.428147871	0.147	0.164	6.98 × 10^−6^
Kdelr1	2.02 × 10^−8^	−0.324198854	0.284	0.297	7.21 × 10^−6^
Trbc2	2.75 × 10^−8^	−0.285374632	0.618	0.675	9.82 × 10^−6^
Il17f	5.21 × 10^−8^	2.059823745	0.073	0.029	1.86 × 10^−5^
Klrg1	7.89 × 10^−8^	0.264761374	0.576	0.594	2.82 × 10^−5^
Junb	7.97 × 10^−8^	−0.388272967	0.31	0.325	2.85 × 10^−5^
Nlrp3	1.26 × 10^−7^	−0.597921116	0.153	0.166	4.51 × 10^−5^
Myc	2.41 × 10^−7^	−0.476616459	0.218	0.253	8.62 × 10^−5^
Arl4c	3.15 × 10^−7^	−0.343818516	0.341	0.361	0.00011248
Gimap7	5.57 × 10^−7^	0.317868428	0.203	0.135	0.00019883
Cnot2	6.58 × 10^−7^	−0.286063488	0.305	0.305	0.00023478
Irf3	7.31 × 10^−7^	−0.34329121	0.212	0.212	0.00026102
Rgs1	7.45 × 10^−7^	−0.293196074	0.932	0.907	0.0002659
Bcl2a1a	1.31 × 10^−6^	0.367564466	0.389	0.302	0.00046852
Tlr1	1.62 × 10^−6^	−0.421900744	0.175	0.19	0.00057817
Top2a	5.29 × 10^−6^	−0.72772586	0.187	0.208	0.00189025
Pask	1.41 × 10^−5^	−0.429494104	0.022	0.051	0.0050418
Slc25a37	1.88 × 10^−5^	−0.484839473	0.085	0.118	0.00672856
Cd28	2.02 × 10^−5^	0.347323381	0.051	0.021	0.00721928
Tnfsf10	2.27 × 10^−5^	−0.348889171	0.109	0.1	0.00809477
Il13	2.49 × 10^−5^	−0.856489991	0.212	0.252	0.00888811
2810417H13Rik	2.70 × 10^−5^	−1.084685083	0.098	0.139	0.00963187
Ltb	6.02 × 10^−5^	−0.619564112	0.2	0.258	0.02147622
Aurkb	0.00010033	−0.606683414	0.068	0.08	0.03581788
Cd2	0.000109757	0.874849673	0.087	0.049	0.03918321
Thy1	0.000114649	0.303535708	0.529	0.595	0.04092978
Cd79b	0.000122918	−0.567101439	0.081	0.115	0.04388156
Dusp2	0.000155975	0.600080716	0.101	0.061	0.05568313
Lamp3	0.000168547	−0.30460207	0.055	0.065	0.06017141
Trac	0.000224842	0.355390225	0.085	0.051	0.08026863
Nt5e	0.000261042	−0.439126095	0.187	0.211	0.09319203
Casp1	0.000291689	−0.409766052	0.216	0.25	0.10413292
Kit	0.000310008	−0.476509702	0.186	0.219	0.11067269
Il15ra	0.000401729	−0.281791311	0.25	0.221	0.14341725
Il10	0.000479278	−0.933714545	0.076	0.11	0.1711022
Traf6	0.000551735	−0.509127939	0.085	0.102	0.19696928
Ube2c	0.001764614	−0.911248134	0.058	0.089	0.62996736
Tlr2	0.002256214	−0.41597756	0.058	0.073	0.80546847
Mki67	0.003036629	−0.591176811	0.066	0.095	1
Cd63	0.008035721	−0.338608731	0.122	0.154	1
Irf4	0.07262249	−0.296611495	0.052	0.063	1
Pik3ip1	0.204664446	−0.275481541	0.058	0.071	1
Ifitm2	0.344980272	0.491085589	0.078	0.068	1

Genes associated with the ILC2 phenotype are reduced with *ex vivo* E2 treatment in males and females.

**Figure 5:**
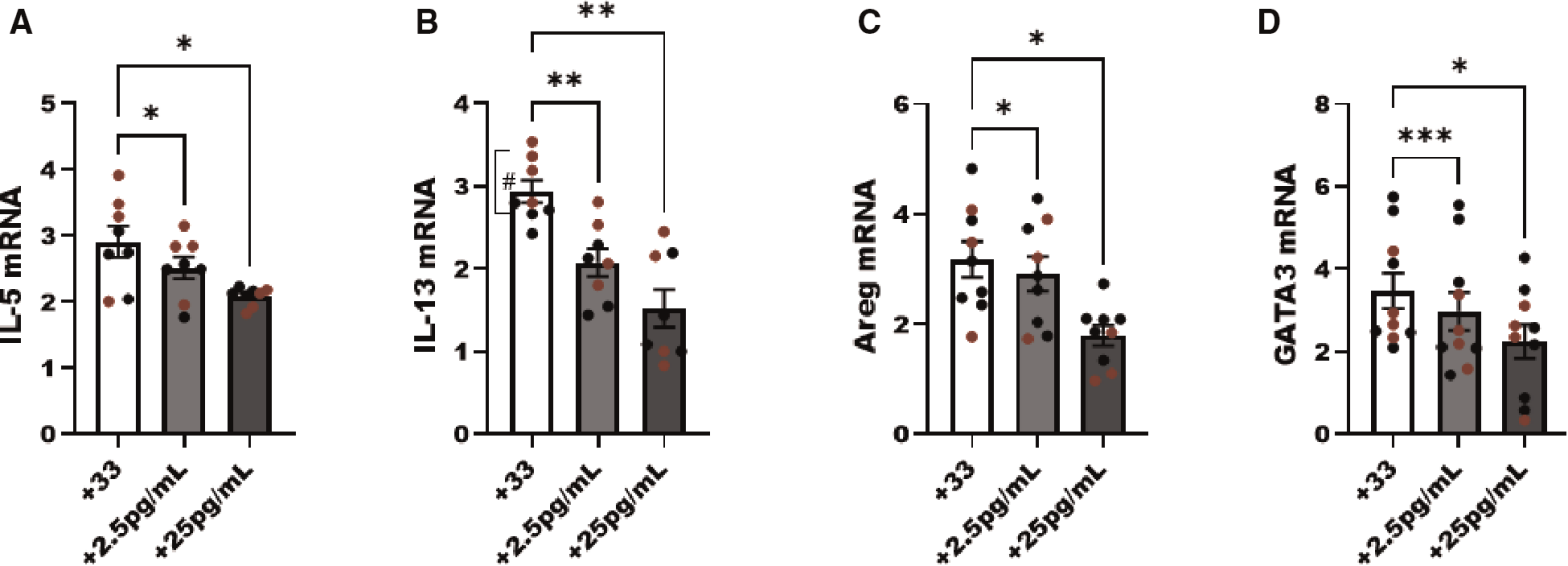
Genes associated with ILC2 phenotype are reduced with estrogen treatment in males and females. Male (black circles) and female (maroon circles) ILC2 were sorted from the lungs and cultured with titrated amounts of E2 and IL-2 and IL-7 with IL-33 for 5 days. (**A**) *il-5*, (**B**) *il-13*, (**C**) *areg* and (**D**) *gata3* mRNA expression was quantified *via* qRT-PCR. Error bars denote means ± SEM (*n* = 8–10 mice/group). Data from two experiments are shown. Groups were compared using two-way ANOVA and Tukey's multiple comparison test. *p < 0.05; **p < 0.01; ***p < 0.001; ****p < 0.0001 (“#” symbolizes comparison of male vs. female group using Mann-Whitney test, #*p* = 0.028).

In the next studies we used untreated male and female animals to compare the regulation of NF-κB without an allergen challenge to interpret the effects of estrogen in ILC2 directly without *in vivo* exposure to pro-allergy cytokines (i.e., IL-33) complicating the analysis. We hypothesized that physiological doses of E2 (2.5 and 25 pg/ml) would disrupt the IL-33 responses (i.e., IL-5, and IL-13 production). Initially, we performed E2 titration experiments to determine whether estrogen itself could activate ILC2 and whether estrogen at a higher concentration would decrease viability. As such we started our experiments with E2 at a high dose of 250 pg/ml, and performed 10-fold serial dilutions down to 0.25 pg/ml of estrogen. Cell viability was measured in every experiment to show that even the highest dose of E2 did not reduce ILC2 viability after 5 days of culture (data not shown). We included non-estrogen treated male and female ILC2 to keep our range of biological sex differences in mind as we evaluated differences in cytokine transcripts following IL-33 stimulation. A low (2.5 pg/ml) and moderate dose (25 pg/ml) of E2 reduced *IL-5*, *IL-13*, *Areg* and *GATA-3* transcripts in male and female ILC2 compared to non-estrogen treated groups (Figures 5A–D). IL-13 transcripts were higher in females compared to males, as previously shown (#*p* < 0.05) (14) (Figure 5B).

NF-κB pathway associated genes are sensitive to E2 treatment in both male and female ILC2.

**Figure 6:**
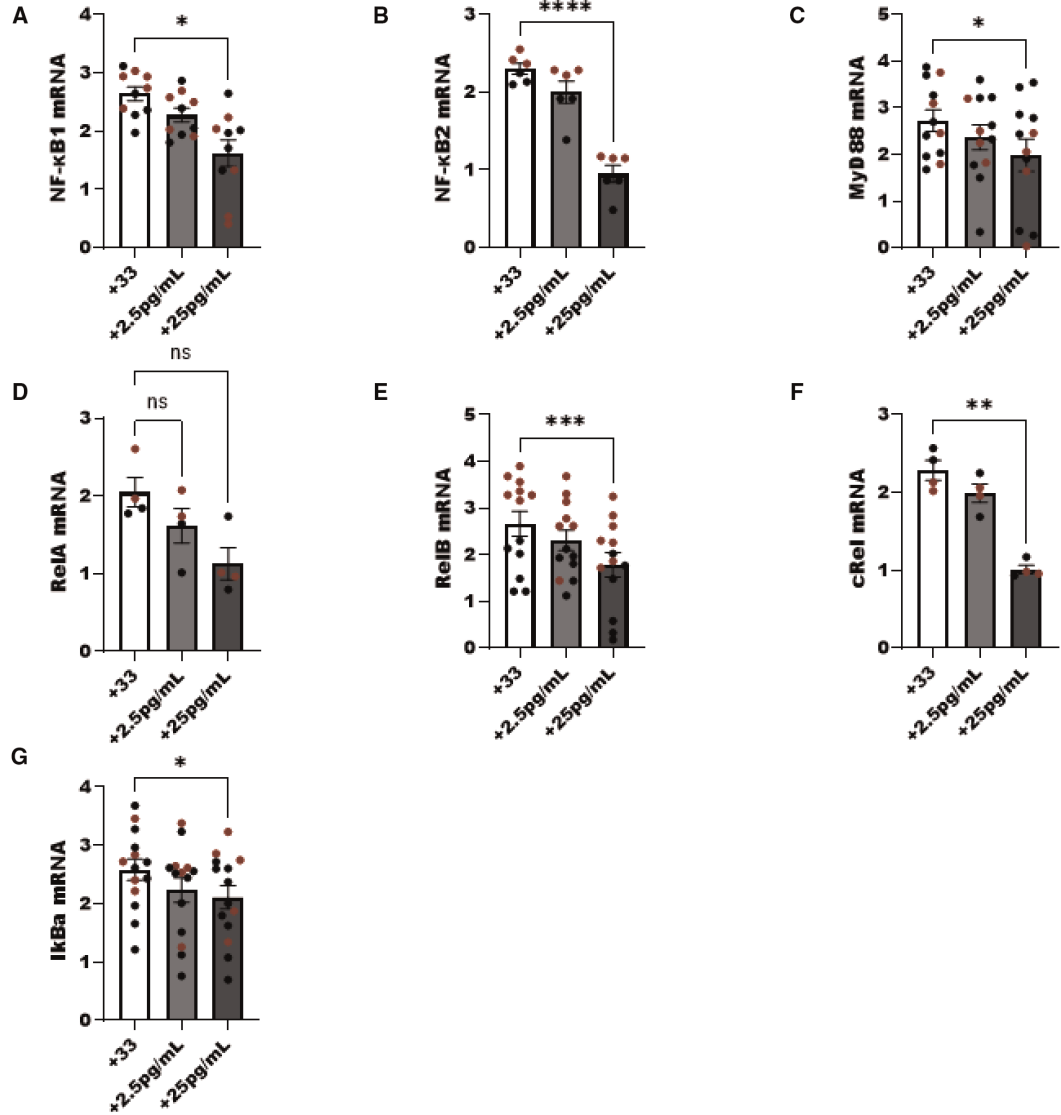
NF-κβ pathway components are reduced in ILC2 by E2 treatment. Male (black circle) and female (maroon circle) ILC2 were sorted from the lungs and cultured with titrated amounts of E2 and IL-2 and IL-7 with IL-33 for 5 days. (**A–G**) Components of the NF-κB pathway (**A**) *NFκβ1 (p50)* (**B**) *NFκβ2 (p52)*, (**C**) *MyD88*, (**D**) *relA*, (**E**) *relB*, (**F**) *cRel* and (**G**) *IκBa* mRNA were evaluated for responsiveness to low 2.5 pg/ml and moderate dose (25 pg/ml) estrogen in combination with IL-33 treatment. Groups were compared using one-way ANOVA and Tukey's multiple comparison test. *p < 0.05; **p < 0.01; ***p < 0.001; ****p < 0.0001.

We noted a dose dependent decrease in NF-κB pathway components following increasing E2 to 25 pg/ml in culture. NFκB1 (p50), MyD88, cRel and the inhibitor of NF-κB (IkBa) decreased as the level of E2 increased (*p* < 0.05). Components of the non-classical NFκB pathway were examined as well, and both NF-κB2 and RelB were significantly decreased with the addition of E2 (*p* < 0.05) (Figure 6).

## Discussion

Stressed, hyper-reactive, or damaged airways trigger the release of IL-33 which activates the innate lymphoid program to elicit an allergic asthma lung phenotype. IL-33 is a potent cytokine for producing ILC2 expansion in the lung, and we have noted significant responses of isolated ILC2 to IL-33 in combination with IL-2 and IL-7 *ex vivo*. Previous studies have suggested that female sex hormones contribute to the sex differences in allergic asthma. Han et al. showed in the fully adjusted model that in obese women, higher serum estradiol and serum free testosterone were, respectively, associated with a 40% and a 40%–50% reduction in the odds of current asthma (39). Estrogen has been shown to regulate macrophages, dendritic cells, eosinophils and mast cells in asthma (40), but how estrogen regulates lung ILC2 in asthma has not been studied. For this study we evaluated the effects of estrogen on the systemic response to allergen mediated by the NF-κB pathway. Although studies with ovariectomized mice have demonstrated that numbers of lung ILC2 are not affected by levels of estrogen (41, 42) and are not in agreement with our current findings, it is important to note that these studies were done in steady state (42) and OVX mice received both E2 and progesterone pellets (unlike ours where OVX mice received only E2) (41). We believe that progesterone has the opposite effects of estrogen on ILC2 responses, and we have observed an increased allergic inflammatory response in OVX mice that received only progesterone pellets (unpublished). None of these studies used OVA sensitization and challenge model. The present study identified the role of estrogen in modulating canonical as well as non-canonical NF-κB family member's activity in ILC2 and reducing IL-5 and IL-13 cytokine production in ILC2.

NF-κB is a master regulator of inflammatory response and NF-κB pathway can be activated by a broad number of cytokine and physiologic mediators, including IL-33, nitric oxide, reactive oxygen species, and antigen specific responses of B cells and T cells. In our study addition of E2 reduced canonical and non-canonical components of NF-κB pathway. The non-canonical NF-κB pathway has been shown to regulate TNF-α induced TNFR2 ILC2 signaling in allergic asthma (43). We also measured phosphorylation of the p65 subunit (RelA) using flow cytometry and examined multiple classical and non-classical NF-κB pathway components by qRT-PCR. Our results confirmed that estrogen had a potent effect on reducing aspects of the NF-κB pathway leading to decreased IL-5. Other groups have shown that *Alternaria*-induced asthma increases the extent of cytokine production in females compared to males. Our sc-RNA seq results showing differential expression of genes in males and females are consistent with previously described sex-bias in asthma and showed involvement of NF-κB transcription factors. We found interferon regulatory factor 7 (IRF7) was upregulated in male murine lung ILC2 compared to females. ILC2 from asthma patients have shown higher level of IRF7 than those from healthy donors and IRF7 expression was also reported in murine lung ILC2 upon papain or IL-33 stimulation (44). Data indicated significant upregulation of vascular associated smooth muscle cell proliferation in untreated females compared to males. It has been shown before that in human airway smooth muscle clinically relevant concentrations of exogenous estrogen facilitates bronchodilation (45). This suggests estrogen and NF-κB pathway components as potentially novel therapeutic target in airway hyper responsiveness.

Compared to male ILC2, female ILC2 (clustered in 1, 4 and 6) expressed increased transcripts encoding cytokine and cytokine related genes (for example, *il13*, *il10*), transcriptional regulators (for example, *fosb*, *jun*, *egr1*), nuclear factors (for example, *nrp1*), and enzymes (for example, Dusp1). Although heterogeneity in ILC2 is apparent among different tissues (46), here we highlight the heterogeneity in male and female lung-resident ILC2 in asthma. Future studies should determine whether these sex-specific expression profiles are influenced by hormones and are acquired during fetal or perinatal development when ILC2 become established in tissues (47, 48). Study in pregnant women suggests a role of sex hormones in influencing circulating ILC2. The proportion of circulating ILC2 were significantly higher in late-pregnant women, than early-pregnant or non-pregnant women, and were correlated with elevated plasma estradiol and progesterone levels (49). The direct evidence of sex hormones regulating ILC2 and effector cytokine secretion in asthma patients is worth of exploring.

The limitations of our study is that firstly other than p65 we did not evaluated protein expression of other NF-κB pathway components. Secondly, we could not efficiently measure estrogen receptor α and estrogen receptor β and G Protein-Coupled Receptor in ILC2 obtained from ovarian hormone treated ovariectomized BALB/c mice because these cells were rarer than female-sham treated mice. Furthermore, we used a mouse immune response targeted panel which lacked estrogen receptor genes and did not perform whole transcriptome analysis. Others have reported that lung ILC2 express lower levels of estrogen receptor α and estrogen receptor β in comparison to the androgen receptor, but this was only shown as RNA sequencing data and not reported at the protein level in these cells. It is known that estrogen signaling is predominantly mediated by ERα and ERβ receptors in other cells but certainly needs to be confirmed at the protein level in ILC2 (50). It is possible that these receptors get upregulated in ILC2 following ovarian hormone treatment, it has been shown that hormone receptor expression in circulating ILC2 change in non-pregnant and pregnant women indicating the association of estrogen and progesterone with ILC2 in human peripheral blood (49). Alternatively, estrogen can induce cytoplasmic activation of NF-κB (21, 51). Thirdly, NF-κB can act as a homodimer (e.g., c-Rel/c-Rel), and/or a heterodimer (e.g., c-Rel/p50) and further evaluation of downstream protein expression and characterization of functional roles in different combinations of NF-κB members using NF-κB inhibitors in estrogen treated ILC2 is needed. Lastly, among mitogen-activated protein kinase (MAPK) family members, p38 MAPK subgroup is most involved in airway and lung inflammation underlying asthma and p38 MAPK signaling has shown to regulate cytokine production in IL-33 stimulated ILC2 (52, 53), effect of estrogen on MAPK family of kinases (p38, ERK 1/2, and JNK and PI3-K) in ILC2 warrants investigation.

Low doses of estrogen may be an effective means for reducing asthma exacerbations in asthmatic men and women. Given the fluctuations in estrogen during normal cycling (i.e., low levels at time of menstruation) was suggested more than a decade ago as mechanism for airway hyper reactivity (12, 54, 55), if low doses of hormones are given this could restore normalcy in the airways to reduce bronchoconstriction, this in turn could prevent long term stays in the hospital due to uncontrolled asthma exacerbations. In conclusion, our results suggests that estrogen can play a protective role in allergic asthma by negatively regulating IL-33 induced ILC2 and IL-5/IL-13 cytokine production, furthermore we demonstrated that this effect is regulated by NF-κB signaling pathway. Understanding the signaling cascades activated by estrogen in ILC2 is critical in asthma, as elucidation of the molecular actions will lead to better therapeutics.

## Data Availability

The original contributions presented in the study are included in the article/Supplementary Material, further inquiries can be directed to the corresponding author/s.
